# Donor-derived cell-free DNA testing for noninvasive monitoring of allograft rejection after lung transplantation

**DOI:** 10.3389/fimmu.2026.1895278

**Published:** 2026-07-29

**Authors:** Shuo Ding, Yiyang Sun, Xiangxin Zheng, Shixiong Mai, Jing Wu, Yongshan Xu, Jingyu Chen, Man Huang

**Affiliations:** 1 Department of General Intensive Care Unit, The Second Affiliated Hospital of Zhejiang University School of Medicine, Hangzhou, China; 2 Key Laboratory of Multiple Organ Failure (Zhejiang University), Ministry of Education, Hangzhou, China; 3 Lung Transplantation Center, The Second Affiliated Hospital of Zhejiang University School of Medicine, Hangzhou, China; 4 Binjiang Institute of Zhejiang University, Hangzhou, China

**Keywords:** clinical rejection, donor-derived cell-free DNA, lung transplantation, noninvasive monitoring, single lung transplantation

## Abstract

**Background:**

Donor-derived cell-free DNA (dd-cfDNA) is a promising noninvasive marker of graft injury after lung transplantation (LTx), but its real-world value for identifying rejection-associated allograft injury and its interpretation across infection status and transplant procedures remain uncertain.

**Methods:**

This single-center retrospective study consecutively included LTx recipients undergoing inpatient follow-up and dd-cfDNA testing from February 2024 to March 2026. Analyses used observations obtained ≥1 month after LTx. Diagnostic performance was assessed by ROC analysis, and the association between dd-cfDNA (%) and clinically diagnosed rejection was evaluated by multivariable logistic regression. Single- and double-LTx recipients were compared, and a stable-state correction factor was explored.

**Results:**

Among 161 recipients, 218 dd-cfDNA tests were performed; 196 non-perioperative observations were analyzed, including 64 rejection and 132 non-rejection observations. Both dd-cfDNA (%) and dd-cfDNA (copies/mL) were higher in rejection. dd-cfDNA (%) yielded an AUC of 0.677 (95% CI, 0.600-0.755), with a cutoff of 1.03% (sensitivity, 90.6%; specificity, 40.9%). dd-cfDNA (copies/mL) yielded an AUC of 0.666 (95% CI, 0.584-0.748), with a cutoff of 104.10 copies/mL (sensitivity, 48.4%; specificity, 78.8%). dd-cfDNA (%) remained independently associated with rejection (adjusted OR, 1.36; 95% CI, 1.15-1.62; P<0.001). Single-LTx recipients had lower dd-cfDNA (%) than double-LTx recipients under stable and rejection conditions; this difference was attenuated after correction (CF = 1.54).

**Conclusions:**

dd-cfDNA may serve as a noninvasive adjunct for identifying rejection-associated allograft injury after LTx. Infection and transplant procedure should be considered during interpretation, and stable-state correction may improve comparability between single- and double-LTx recipients.

## 1 Introduction

Over the past two decades, the volume of lung transplantation (LTx) has increased substantially, with nearly 6,000 procedures performed in China since 2015 (1). Despite improvements in donor-graft use, operative strategies, and peri-transplant care, long-term outcomes after LTx remain less favorable than those after other solid organ transplants, with reported 1-, 5-, and 10-year survival rates of 89.4%, 61.2%, and 33.1%, respectively (2–4).

Graft-related complications, particularly immune rejection, remain major barriers to long-term survival and quality of life after LTx (5–9). At present, the diagnosis of rejection after LTx still relies on an integrated assessment of clinical manifestations, decline in lung function, imaging findings, and immunologic evidence (7, 8). Histopathology remains the diagnostic gold standard. However, tissue biopsy is invasive, carries risks such as pneumothorax and bleeding, and may be difficult to repeat in older or clinically fragile recipients. In real-world practice, patient preference, procedural risk, sampling error, and interobserver variability further limit its use for longitudinal monitoring. Therefore, a repeatable noninvasive biomarker that complements routine clinical assessment is needed (10–14).

Donor-derived cell-free DNA (dd-cfDNA), released into recipient blood after donor-cell injury, apoptosis, or necrosis, has shown promise for monitoring graft injury after solid organ transplantation. In LTx, retrospective studies have reported higher dd-cfDNA levels during rejection than non-rejection states (15–19). However, dd-cfDNA reflects graft injury rather than rejection specifically, and its interpretation may be influenced by infection, systemic inflammation, and testing time. Infection can mimic rejection clinically and may affect dd-cfDNA specificity. Besides, single- and double-LTx differ in graft mass and donor-DNA release, which may influence baseline levels and diagnostic thresholds. Data supporting standardized interpretation in Asian populations remain limited (20–24).

Therefore, this study evaluated the diagnostic and interpretive value of dd-cfDNA in a large Asian LTx cohort. We compared dd-cfDNA (%) and absolute concentration (copies/mL) for detecting rejection-associated graft injury, assessed the association between dd-cfDNA (%) and clinically diagnosed rejection after adjustment for key confounders, explored the influence of single- versus double-LTx, and examined prespecified clinical subgroups. The findings of this study may provide important evidence to support the individualized and precise use of dd-cfDNA in the management of LTx recipients.

## 2 Materials and methods

### 2.1 Study design and study cohort

This was a single-center, retrospective observational study. We included LTx recipients who underwent inpatient follow-up and dd-cfDNA testing at the Lung Transplant Center of the Second Affiliated Hospital of Zhejiang University School of Medicine between February 2024 and March 2026. Perioperative cases, defined as those within 1 month after LTx, were included only in the descriptive analysis and were excluded from the primary diagnostic analysis. Because some recipients underwent more than one dd-cfDNA test during follow-up, the primary unit of analysis was the dd-cfDNA testing event. Patient-level clustering was addressed in sensitivity analyses using generalized estimating equations and by restricting the dataset to one eligible observation per recipient (**Supplementary Figure 1**).

The inclusion criteria were as follows: patients who had undergone LTx; had at least one postoperative dd-cfDNA test; and had available clinical and laboratory data that could be retrospectively reviewed.

The exclusion criteria were death within 30 days after LTx, receipt of another solid organ transplant or combined organ transplantation, retransplantation before dd-cfDNA measurement, and substantial missing key clinical information that precluded the primary analysis.

The study protocol was approved by the Ethics Committee of the Second Affiliated Hospital of Zhejiang University School of Medicine (approval No. IRB-2024-0016). Because this study was based on retrospective data analysis, the requirement for informed consent was waived.

### 2.2 Data sources and variable collection

Study data were obtained from the institutional LTx database and electronic medical record system. Data were collected through structured extraction and manual verification. The following demographic and transplant-related variables were collected: age, sex, body mass index (BMI), primary disease, baseline comorbidities, history of HSCT, surgical procedure, date of transplantation, anti-rejection regimen, and immunosuppressant metabolism profile. Clinical and laboratory variables included dd-cfDNA results, time from LTx to testing, lymphocyte count, panel reactive antibody (PRA), histopathologic evidence, CMV and EBV nucleic acid test results, and information on bacterial, fungal, and other viral infections. dd-cfDNA was measured using a commercially available next-generation sequencing (NGS)-based platform, *AlloDx*, and testing was performed independently by the central laboratory. 10 mL of peripheral venous blood was collected from each recipient, followed by plasma separation and extraction of total plasma cfDNA. dd-cfDNA was quantified using an Illumina-based high-throughput sequencing platform targeting approximately 5,800 single nucleotide polymorphism (SNP) loci in plasma cfDNA. dd-cfDNA (%) was calculated according to the proportion of donor-derived SNP signals relative to total cfDNA. The absolute concentration was derived from the same NGS-based analytical pipeline and does not rely on digital PCR. The analytical lower limit of detection for dd-cfDNA (%) was 0.3%. Quality control included sequencing-based depth and SNP coverage metrics inherent to the AlloDx bioinformatics pipeline. In this study, the reference standard for evaluating the performance of dd-cfDNA was the integrated clinical diagnosis of acute or chronic rejection, based on all available clinical, imaging, and laboratory data.

### 2.3 Research definition and grouping

The primary endpoint was clinically diagnosed rejection-associated allograft injury, it was determined according to relevant ISHLT consensus recommendations and the clinical practice of our center. The adjudication was performed by experienced lung transplant physicians based on an integrated assessment of respiratory symptoms, changes in oxygenation and lung function, radiological findings, immunological evidence including PRA and donor-specific antibody information, microbiological results, response to anti-rejection treatment, and histopathological findings when available (6, 10). As dd-cfDNA reflects allograft injury rather than rejection specifically, alternative causes of graft dysfunction were considered during clinical adjudication, including bacterial, fungal, or viral infection, airway complications, ischemia-reperfusion-related injury, drug toxicity, chronic lung allograft dysfunction (CLAD), surgical complications, and other non-alloimmune processes. Cases were classified as clinically diagnosed rejection only when the overall clinical evidence supported rejection-associated allograft injury after consideration of these alternative explanations.

Given that in this retrospective real-world cohort, the biopsy time was not protocolized and was influenced by patient condition, procedural risk, and clinical feasibility, histopathological confirmation was defined as rejection confirmed by transbronchial biopsy within 3 months before or after dd-cfDNA testing, or by explant pathology at subsequent retransplantation. Infection status was defined as positive when any bacterial, fungal, or viral pathogen, including HCMV or EBV, was detected by metagenomic NGS or confirmed by repeated body fluid cultures. Such cases were classified as infection-positive.

### 2.4 Single/double LTx dd-cfDNA correction analysis

To assess the effect of surgical procedure on the interpretation of dd-cfDNA, dd-cfDNA levels were compared between single- and double-LTx recipients under both stable conditions, defined as no rejection, and clinical rejection. A correction factor (CF) was derived from the ratio of the median dd-cfDNA (%) value in double-LTx recipients to that in single-LTx recipients within the stable cohort. The original dd-cfDNA (%) values in single-LTx recipients were then multiplied by this CF (21). After correction, between-group comparisons, receiver operating characteristic (ROC) analysis, and assessment of clinical net benefit were repeated.

### 2.5 Subgroup analysis and sensitivity analysis

Eight clinical subgroups were prespecified: surgical procedure, age, solid tumor, HCMV-DNA status, infection status, immunosuppressant metabolism profile, history of HSCT, and rheumatic disease. The purpose of the subgroup analyses was not to replace multivariable adjustment, but to assess the consistency and heterogeneity of the primary findings across different clinical settings, identify potential effect modifiers, and inform the development of future models. An additional exploratory analysis was performed using three mutually exclusive groups—stable, infection, and rejection—to examine the potential effect of infection itself on dd-cfDNA levels.

### 2.6 Statistical analysis

Statistical analyses were performed using SPSS 26.0 and R software (version 4.4.1; R Foundation for Statistical Computing, Vienna, Austria). ROC curve analysis was used to evaluate the performance of dd-cfDNA in detecting rejection-associated graft injury, and the optimal cutoff value was determined using the Youden index. Because some recipients contributed more than one dd-cfDNA testing event, sensitivity analyses were performed using generalized estimating equations with patient-level clustering and robust standard errors. An additional sensitivity analysis was performed by retaining only the first eligible non-perioperative observation from each recipient. Multivariable logistic regression, with multicollinearity assessed by variance inflation factors and model performance (discrimination by AUC, calibration by Brier score) evaluated via bootstrap internal validation, was performed to assess the independent association between dd-cfDNA (%) and rejection-associated graft injury after adjustment for age, time from LTx to testing, surgical procedure, infection status, and history of HSCT. The clinical utility of single-LTx correction was assessed using two single-marker logistic models based on original and CF-corrected dd-cfDNA (%). Clinical net benefit before and after CF correction was assessed using decision curve analysis (DCA) across clinically relevant threshold probabilities. Reclassification improvement was evaluated using the continuous net reclassification improvement (NRI) index. Event NRI, non-event NRI, and total NRI were reported separately. Their 95% confidence intervals were estimated using bootstrap resampling at the level of dd-cfDNA testing events. In each bootstrap sample, the CF was rederived and both the uncorrected and corrected single-marker logistic regression models were refitted. All tests were two-sided, and P<0.05 was considered statistically significant.

## 3 Results

### 3.1 Study population and overall temporal distribution

**Figure 1:**
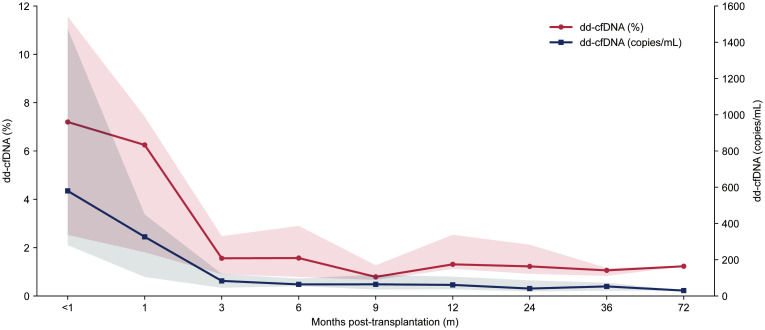
Temporal trends of dd-cfDNA at selected clinically relevant follow-up time points after transplantation. Lines represent median values, and shaded areas indicate the interquartile range (Q25-Q75). The horizontal axis only shows the follow-up time points of clinical concern. “<1” indicates measurements obtained within 1 month after transplantation.

**Figure 2:**
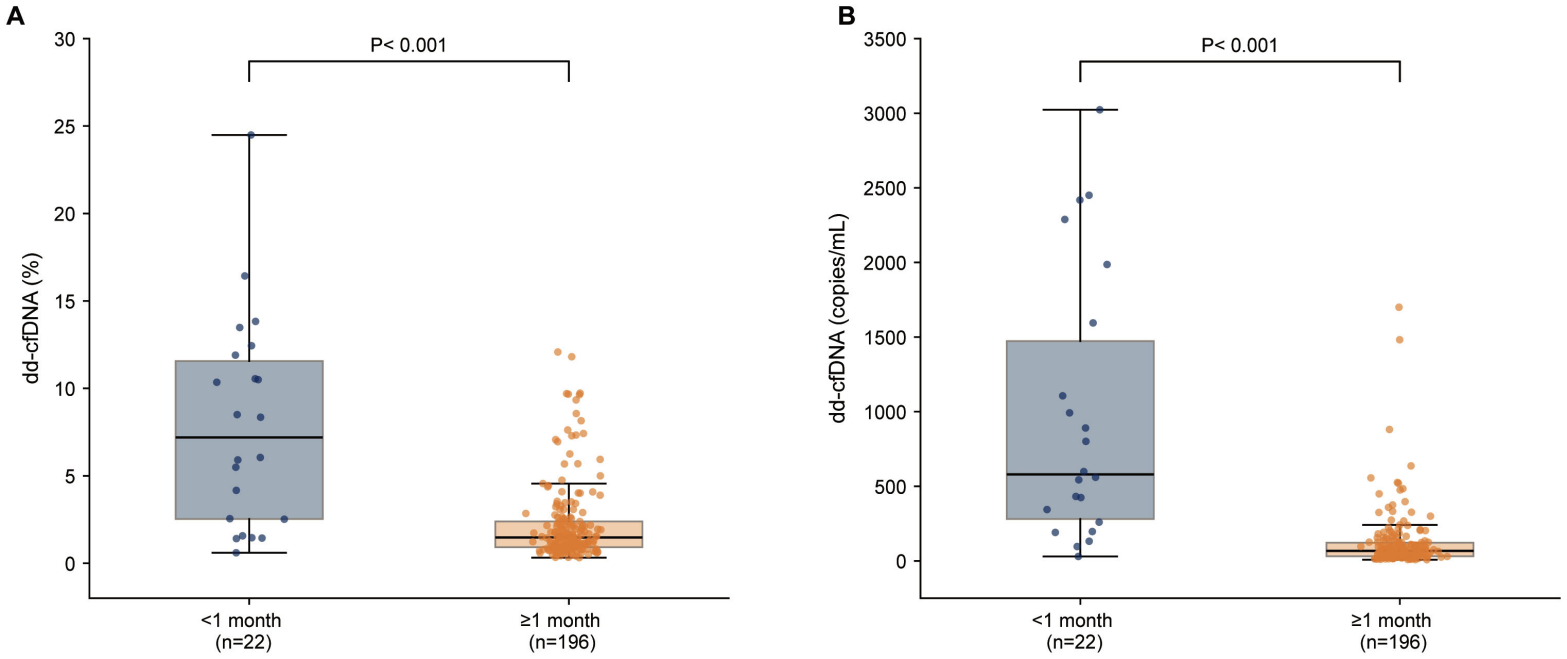
Comparison of **(A)** dd-cfDNA (%) and **(B)** dd-cfDNA (copies/mL) between perioperative and non-perioperative groups.

A total of 161 LTx recipients were included, with 218 dd-cfDNA testing events. Of these, 22 tests were performed during the perioperative period, defined as within 1 month after LTx, and 196 were performed at 1 month or later after LTx. The temporal distribution showed that dd-cfDNA levels were markedly elevated in the early postoperative period, followed by a rapid decline and maintenance at relatively low levels during follow-up (**Figure 1**). In the overall cohort, the median dd-cfDNA (%) was 1.52 [0.93, 2.89], and the median dd-cfDNA (copies/mL) was 78.59 [35.26, 156.89]. dd-cfDNA (%) was significantly higher in the <1-month group than in the ≥1-month group (7.20 [2.53, 11.56] vs. 1.48 [0.92, 2.39]; Mann-Whitney U test, P<0.001). Similarly, dd-cfDNA (copies/mL) was significantly higher in the <1-month group than in the ≥1-month group (579.86 [280.85, 1472.78] vs. 67.88 [31.80, 122.04]; Mann-Whitney U test, P<0.001) (**Table 1**; **Figure 2**). Because perioperative injury itself can substantially increase dd-cfDNA levels, all subsequent primary diagnostic analyses were restricted to the cohort tested at least 1 month after LTx.

**Table 1:** Comparison of dd-cfDNA between perioperative and non-perioperative groups.

Outcomes	All patients (n = 218)	<1 month (n = 22)	≥1 month (n = 196)	P value
dd-cfDNA (%)	1.52 (0.93-2.89)	7.20 (2.53-11.56)	1.48 (0.92-2.39)	<0.001
dd-cfDNA (copies/mL)	78.59 (35.26-156.89)	579.86 (280.85-1472.78)	67.88 (31.80-122.04)	<0.001

Data are expressed as mean ± standard deviation, median (interquartile range), or *n* (%).

### 3.2 Baseline characteristics of main analysis cohort

After excluding 22 perioperative testing events, defined as those performed within 1 month after LTx, the primary analysis cohort included 196 testing events from 152 patients. Overall, 34 of the 152 recipients (22.4%) contributed repeated measurements, ranging from two to four observations each. The median age at testing was 58 years, and 137 testing events involved male recipients (69.6%). Double-LTx accounted for 146 events (74.5%). Interstitial lung disease was the most common primary indication for LTx (41.8%), followed by chronic obstructive pulmonary disease (18.9%) and bronchiolitis obliterans syndrome (BOS) (17.9%). 154 of 196 observations were infection-positive according to the prespecified definition. Bacterial infection was present in 112 observations, fungal infection in 30, HCMV positivity in 19, and EBV positivity in 85. During the study period, 64 testing events (32.7%) were classified as clinically diagnosed rejection, whereas 132 events (67.3%) were classified as non-rejection. There were no statistically significant differences between the rejection and non-rejection groups in age, sex, BMI, primary disease, surgical procedure, or most comorbidities. Rheumatic disease was more frequent in the non-rejection group than in the rejection group (12.1% vs. 1.6%, P = 0.028). Regarding infection-related variables, the rates of bacterial infection, fungal infection, CMV-DNA positivity, and EBV-DNA positivity did not differ significantly between the two groups. The cumulative infection positive rate in the clinical rejection group was 81.3%, while that in the non-rejection group was 77.3%. Baseline characteristics are shown in **Table 2**.

**Table 2:** Comparison of characteristics between clinical rejection and non-rejection groups.

Characteristics	All patients (n = 196)	Clinical rejection group (n = 64)	Non-rejection group (n = 132)	P value
Age (years)	58.0 (34.8-64.0)	58.0 (44.0-62.0)	58.0 (31.0-64.0)	0.889
Sex, male	137 (69.9)	44 (68.8)	93 (70.5)	0.938
Body mass index (kg/m2)	19.95 ± 3.65	19.53 ± 3.09	20.15 ± 3.89	0.233
Primary diseases				0.550
COPD	37 (18.9)	11 (17.2)	26 (19.7)	
Interstitial lung disease	82 (41.8)	27 (42.2)	55 (41.7)	
Bronchiectasis	5 (2.6)	1 (1.6)	4 (3.0)	
Silicosis	14 (7.1)	7 (10.9)	7 (5.3)	
BOS	35 (17.9)	13 (20.3)	22 (16.7)	
Others	23 (11.7)	5 (7.8)	18 (13.6)	
Comorbidities				
Chronic heart disease	74 (37.8)	23 (35.9)	51 (38.6)	0.835
Solid tumor	18 (9.2)	8 (12.5)	10 (7.6)	0.392
Hematological malignancies/HSCT history	39 (19.9)	15 (23.4)	24 (18.2)	0.501
Rheumatic diseases	17 (8.7)	1 (1.6)	16 (12.1)	0.028
Type of surgery				0.448
Bilateral LTx	146 (74.5)	45 (70.3)	101 (76.5)	
Single LTx	50 (25.5)	19 (29.7)	31 (23.5)	
Anti-rejection regimen				0.603
Dual regimen	64 (32.7)	23 (35.9)	41 (31.1)	
Triple regimen	132 (67.3)	41 (64.1)	91 (68.9)	
PCT (ng/mL)	0.07 (0.05-0.13)	0.08 (0.05-0.19)	0.07 (0.05-0.11)	0.119
CMV-DNA positive	19 (9.7)	5 (7.8)	14 (10.6)	0.717
EBV-DNA positive	85 (43.4)	31 (48.4)	54 (40.9)	0.399
Bacterial infection	112 (57.1)	35 (54.7)	77 (58.3)	0.742
Fungal infection	30 (15.3)	7 (10.9)	23 (17.4)	0.331
Immunosuppressant metabolism type*				0.198
Fast-metabolizer genotype	105 (53.6)	39 (60.9)	66 (50.0)	
Slow-metabolizer genotype	91 (46.4)	25 (39.1)	66 (50.0)	

Data are expressed as mean ± standard deviation, median (interquartile range), or *n* (%).

COPD, Chronic obstructive pulmonary disease;

*Immunosuppressant metabolism type was mainly defined according to CYP3A5 rs776746 genotype test. CYP3A5*1/*1 and CYP3A5*1/*3, corresponding to rs776746 AA and AG genotypes, were classified as fast-metabolizer genotypes, whereas CYP3A5*3/*3, corresponding to the rs776746 GG genotype, was classified as a slow-metabolizer genotype.

### 3.3 Diagnostic value of dd-cfDNA for rejection-associated graft injury

Among the 196 dd-cfDNA quantitative testing events included in the primary analysis, dd-cfDNA (%) was significantly higher in the rejection group than in the non-rejection group (1.92 [1.17, 3.42] vs. 1.21 [0.81, 2.01], P<0.001). Similarly, dd-cfDNA (copies/mL) was significantly higher in the rejection group than in the non-rejection group (95.44 [46.95, 207.63] vs. 55.06 [26.65, 96.86], P<0.001) (**Table 3**).

**Table 3:** Comparison of rejection-related parameters between the clinical rejection and non-rejection groups.

Outcomes	All patients (n = 196)	Clinical rejection group(n = 64)	Non-rejection group (n = 132)	P value
dd-cfDNA (%)	1.48 (0.92-2.39)	1.92 (1.17-3.42)	1.21 (0.81-2.01)	<0.001
dd-cfDNA (copies/mL)	67.88 (31.80-122.04)	95.44 (46.95-207.63)	55.06 (26.65-96.86)	<0.001
Postoperative dd-cfDNA test time (m)	12.0 (6.0-24.0)	12.0 (5.8-28.0)	10.5 (6.0-24.0)	0.414
Lymphocyte count (%)	19.6 (10.4-28.4)	14.7 (7.6-22.2)	21.7 (11.9-31.7)	<0.001
Lymphocyte count (10^9^/L)	1.21 (0.68-1.86)	0.93 (0.51-1.79)	1.27 (0.74-1.92)	0.051
PRA-I	17 (8.7)	8 (12.5)	9 (6.8)	0.185
PRA-II	9 (4.6)	8 (12.5)	1 (0.8)	<0.001
Histopathological confirmation*	18 (9.2)	17 (26.6)	1 (0.8)	<0.001

Data are expressed as mean ± standard deviation, median (interquartile range), or *n* (%).

PRA-I, Panel-reactive antibody class I; PRA-II, Panel-reactive antibody class II.

*Histopathological confirmation was defined as transbronchial biopsy performed within 3 months before or after the dd-cfDNA measurement, or confirmation by explant pathology at retransplantation.

**Figure 3:**
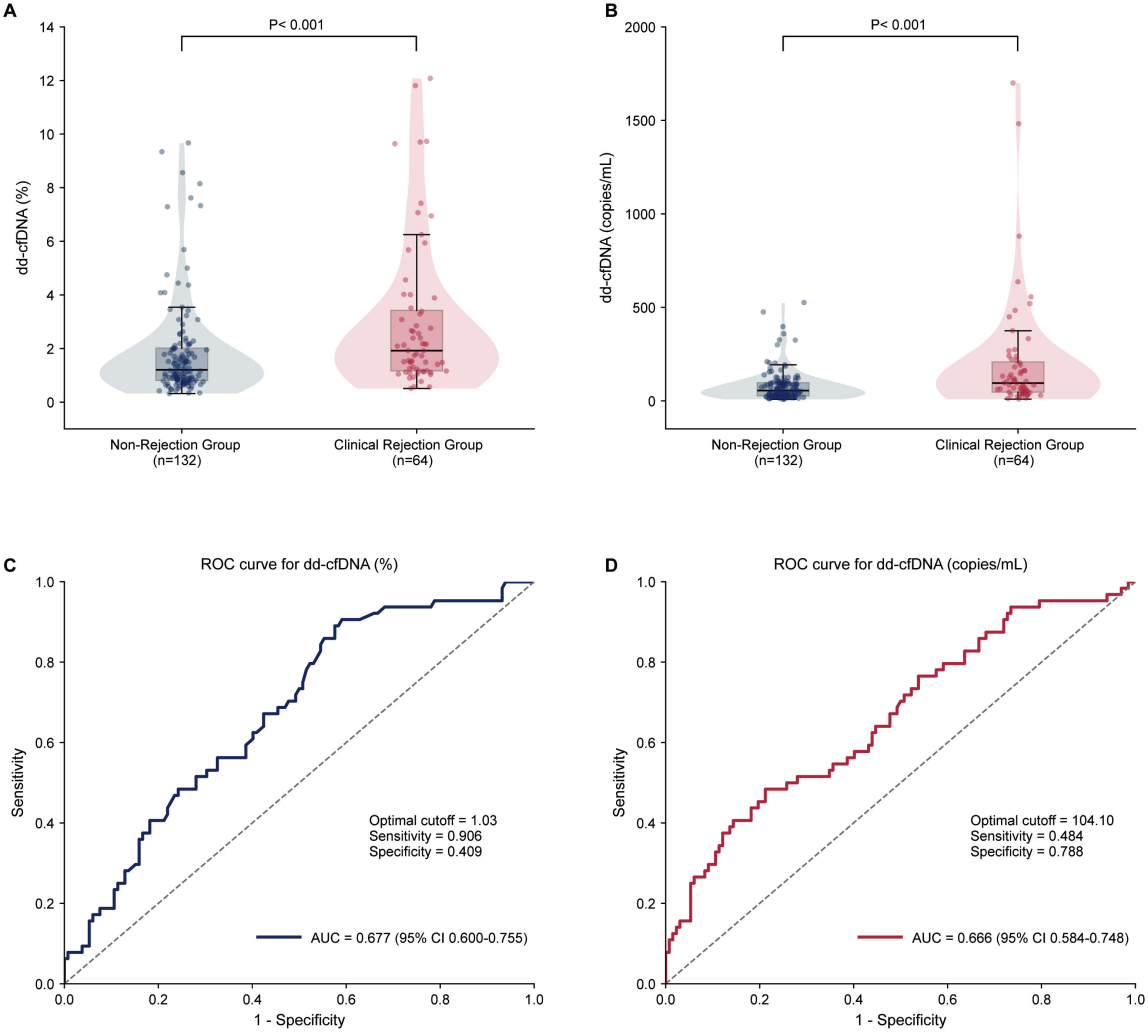
Comparison and diagnostic performance of **(A)** dd-cfDNA (%) and **(B)** dd-cfDNA (copies/mL) between clinical rejection and non-rejection groups, with corresponding ROC analyses shown in **(C, D)**.

ROC analysis showed that for detecting rejection-associated graft injury, dd-cfDNA (%) had an AUC of 0.677 (95% CI, 0.600-0.755), with an optimal cutoff of 1.03% (sensitivity 90.6%, specificity 40.9%; PPV 42.6%, NPV 90.0%; LR + 1.53, LR- 0.23), while dd-cfDNA (copies/mL) yielded an AUC of 0.666 (95% CI, 0.584-0.748) with a cutoff of 104.10 copies/mL (sensitivity 48.4%, specificity 78.8%; PPV 52.5%, NPV 75.9%; LR + 2.28, LR- 0.65) (**Figure 3**). Overall, dd-cfDNA (%) showed a slightly higher AUC and greater sensitivity, suggesting that it may be more suitable as a screening marker. In contrast, dd-cfDNA (copies/mL) demonstrated relatively higher specificity and may provide complementary value for confirming clinical suspicion of rejection.

### 3.4 Independent association between dd-cfDNA and clinically diagnosed rejection and assessment of infection as a confounder

**Figure 4:**
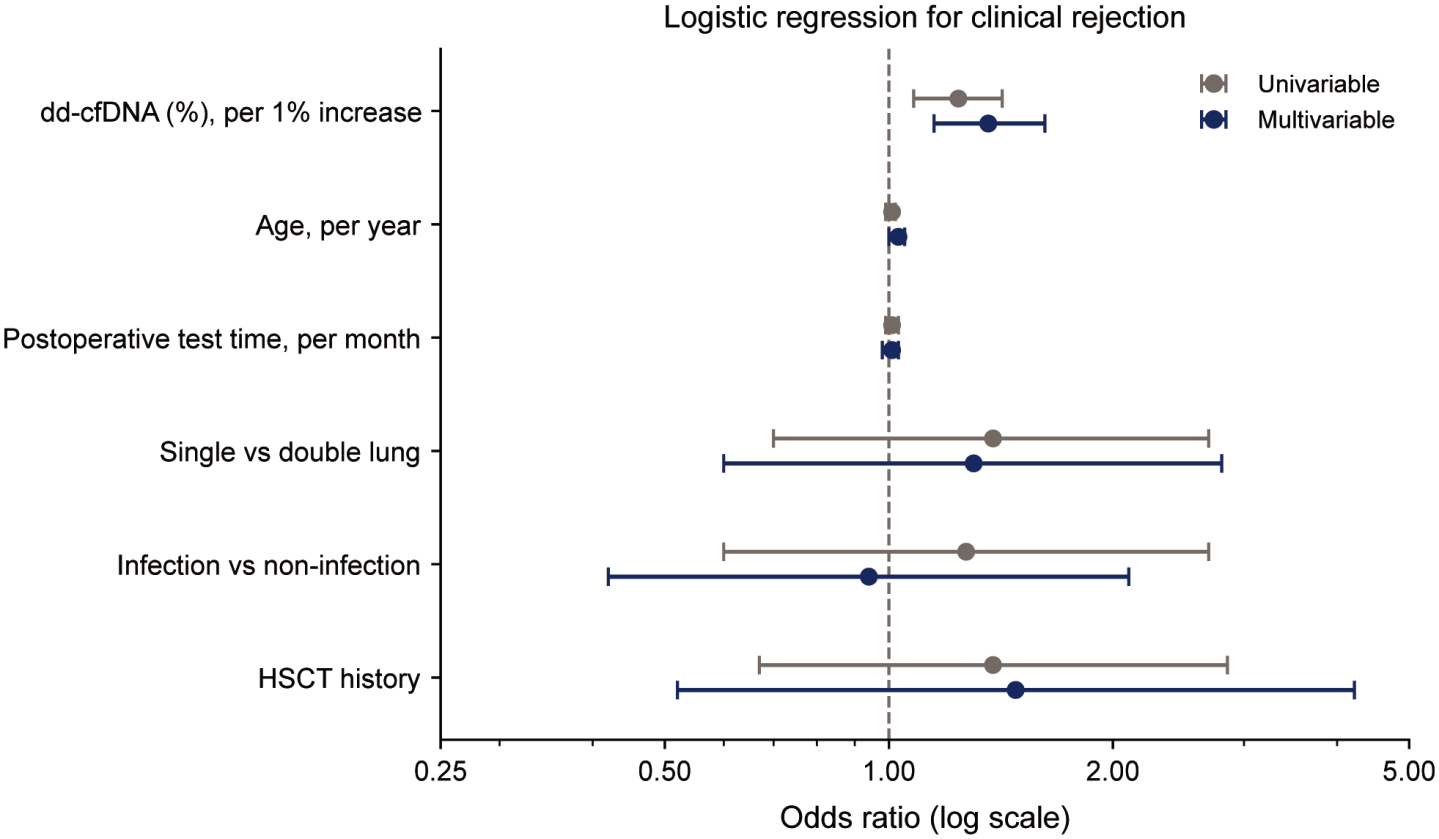
Forest plot of univariable and multivariable logistic regression for clinical rejection. Odds ratios (ORs) and 95% confidence intervals (CIs) are shown for dd-cfDNA (%), age, postoperative dd-cfDNA test time, type of surgery, infection status, and history of HSCT. The multivariable model adjusted for all listed covariates.

A multivariable logistic regression model was constructed to assess whether dd-cfDNA (%) was independently associated with clinically diagnosed rejection after adjustment for age, time from LTx to testing, surgical procedure, infection status, and HSCT history. After adjustment for these factors, dd-cfDNA (%) remained independently associated with clinically diagnosed rejection (adjusted OR, 1.36; 95% CI, 1.15-1.62; P<0.001). Age also showed a weak but statistically significant association with rejection (adjusted OR, 1.03 per year; 95% CI, 1.00-1.05; P = 0.042). In contrast, time from LTx to testing, surgical procedure, infection status, and history of HSCT were not statistically significant in this model (**Table 4**; **Figure 4**).

**Table 4:** Univariable and multivariable logistic regression for clinical rejection.

Variable	Univariable OR (95% CI)	P value	Adjusted OR (95% CI)	P value
dd-cfDNA (%), per 1% increase	1.24 (1.08-1.42)	0.002	1.36 (1.15-1.62)	<0.001
Age, per year	1.01 (0.99-1.02)	0.394	1.03 (1.00-1.05)	0.042
Postoperative test time, per month	1.01 (0.99-1.03)	0.330	1.01 (0.98-1.03)	0.602
Single vs double lung	1.38 (0.70-2.69)	0.351	1.30 (0.60-2.80)	0.511
Infection vs non-infection	1.27 (0.60-2.69)	0.525	0.94 (0.42-2.10)	0.872
HSCT history	1.38 (0.67-2.85)	0.389	1.48 (0.52-4.22)	0.464

Multicollinearity diagnostics did not indicate severe multicollinearity among the covariates. All variance inflation factors were below 3, with values of 1.39 for dd-cfDNA (%), 2.01 for age, 1.11 for postoperative testing time, 1.26 for single versus double LTx, 1.09 for infection status, and 1.68 for HSCT history. The apparent AUC of the multivariable model was 0.693, and the Brier score was 0.199. Bootstrap internal validation using 1,000 resamples yielded an optimism-corrected AUC of 0.647 and an optimism-corrected Brier score of 0.214, further supporting the exploratory interpretation of this model.

To account for repeated dd-cfDNA measurements within the same recipient, a sensitivity analysis using generalized estimating equations with patient-level clustering was performed. The association between dd-cfDNA (%) and clinically diagnosed rejection remained consistent, with an adjusted OR of 1.36 per 1% increase (95% CI, 1.15-1.61; P<0.001). In an additional sensitivity analysis restricted to the first eligible non-perioperative observation from each recipient, 152 recipients and 41 rejection events were included. dd-cfDNA (%) remained significantly associated with clinically diagnosed rejection, with an adjusted OR of 1.63 per 1% increase (95% CI, 1.29-2.05; P<0.001). In this one-observation-per-recipient cohort, the AUC was 0.730 for dd-cfDNA (%) and 0.710 for dd-cfDNA (copies/mL).

Given that concurrent infection is one of the most common competing events after LTx and is biologically plausible as a confounder in the interpretation of dd-cfDNA, we further performed an exploratory analysis using three mutually exclusive groups: Stable, Infection, and Rejection (20, 25). According to the prespecified definitions, the Stable group included cases without rejection or infection (n=30), the Infection group included cases with infection but without rejection (n=102), and the Rejection group included cases with rejection but without infection (n=12). An additional 52 cases had both infection and rejection and were therefore excluded from this mutually exclusive three-group comparison. The median dd-cfDNA (%) values in the Stable, Infection, and Rejection groups were 0.95 [0.70, 1.67], 1.33 [0.85, 2.02], and 2.01 [1.17, 2.17], respectively. Although dd-cfDNA (%) showed an increasing trend across the three groups, the overall difference did not reach statistical significance (Kruskal-Wallis test, P = 0.094). The median dd-cfDNA (copies/mL) values were 53.67 [29.04, 91.51], 60.33 [26.23, 96.89], and 70.09 [34.46, 114.51], respectively, with no significant overall difference among groups (P = 0.699). *Post hoc* pairwise comparisons with Bonferroni correction also showed no significant differences (**Supplementary Figure 2**). These findings suggest that infection may increase the background level of dd-cfDNA. However, because the rejection-only group was small and different infection etiologies were pooled together, the independent contribution of infection burden, pathogen type, and infection severity could not be fully resolved in this retrospective dataset.

### 3.5 Effect of single- versus double-LTx on dd-cfDNA interpretation and correction

Under stable conditions without clinical rejection, the median dd-cfDNA (%) level was significantly lower in single-LTx recipients than in double-LTx recipients (0.93 [0.68, 1.47], n=31 vs. 1.43 [0.85, 2.19], n=101; P = 0.011). A similar difference was observed for dd-cfDNA (copies/mL), with median values of 38.90 [23.36, 53.06] and 74.46 [31.05, 106.46], respectively (P = 0.003). During clinical rejection, dd-cfDNA (%) remained lower in single-LTx recipients than in double-LTx recipients (1.31 [1.14, 2.17], n=19 vs. 2.39 [1.48, 4.01], n=45; P = 0.022). dd-cfDNA (copies/mL) showed the same pattern (52.30 [34.18, 91.59] vs. 126.03 [64.05, 241.68]; P = 0.003).

To account for this systematic difference, a CF for dd-cfDNA (%) was derived from the stable cohort. The CF was calculated as the ratio of the median dd-cfDNA (%) level in double-LTx recipients to that in single-LTx recipients under stable conditions: CF = 1.43/0.93 = 1.54.

After multiplying the original dd-cfDNA (%) values of single-LTx recipients by the CF, the corrected median level in single-LTx recipients under stable conditions was 1.43 [1.05, 2.26], which no longer differed significantly from that in double-LTx recipients (1.43 [0.85, 2.19]; P = 0.475). During clinical rejection, the corrected median value in single-LTx recipients was 2.01 [1.75, 3.33], which was also comparable to that in double-LTx recipients (2.39 [1.48, 4.01]; P = 0.791). These findings suggest that this correction approach improves the numerical comparability of dd-cfDNA (%) between surgical procedures.

Further ROC analysis showed that the diagnostic performance of dd-cfDNA (%) for rejection-associated graft injury was broadly similar between single- and double-LTx recipients. In single-LTx recipients, the AUC of the original dd-cfDNA (%) was 0.688 (95% CI, 0.530-0.845), with an optimal cutoff of 1.08%, a sensitivity of 84.2%, and a specificity of 64.5%. In double-LTx recipients, the AUC was 0.693 (95% CI, 0.604-0.782), with an optimal cutoff of 2.39%, a sensitivity of 51.1%, and a specificity of 77.2%. After CF correction in single-LTx recipients, the AUC remained unchanged at 0.688, whereas the optimal cutoff increased proportionally to 1.66%. This indicates that the primary value of correction lies in improving the comparability of dd-cfDNA values between single- and double-LTx recipients, rather than changing the ranking performance within the single-LTx cohort.

**Figure 5:**
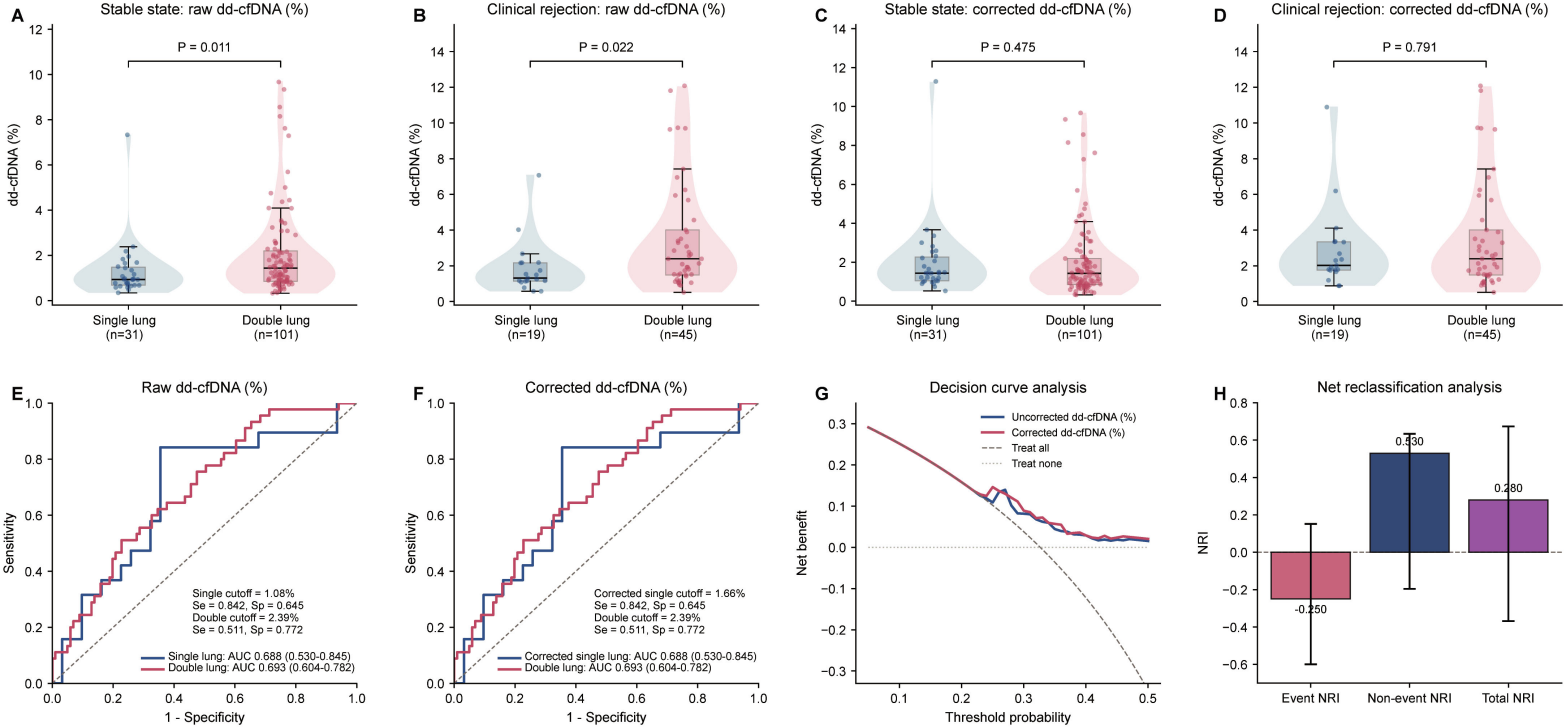
Diagnostic performance and exploratory clinical utility of dd-cfDNA (%) in single- versus double-lung transplant recipients. **(A, B)** Comparison of raw dd-cfDNA (%) between single- and double-lung transplant recipients in the stable non-rejection state **(A)** and during clinical rejection **(B)**. **(C, D)** Comparison after applying a correction factor derived from the stable cohort. **(E, F)** Receiver operating characteristic (ROC) curves for dd-cfDNA (%) in single- and double-lung recipients before **(E)** and after **(F)** correction. The correction factor (CF = 1.54) was calculated as the ratio of median dd-cfDNA (%) between double- and single-lung recipients in the stable state. **(G)** Decision curve analysis comparing the net benefit of corrected and uncorrected dd-cfDNA (%) models across clinically relevant threshold probabilities. **(H)** Continuous net reclassification improvement (NRI) analysis showing event NRI, non-event NRI, and total NRI for the corrected model versus the uncorrected model. Error bars represent 95% confidence intervals estimated by event-level bootstrap resampling, with CF re-estimated and logistic models refitted in each bootstrap sample.

To further assess the potential clinical utility of this correction strategy, single-marker logistic prediction models were constructed using either the original dd-cfDNA (%) or the CF-corrected dd-cfDNA (%). DCA and continuous NRI analyses were then performed. In the combined cohort, the AUC of the uncorrected dd-cfDNA (%) model was 0.677 (95% CI, 0.600-0.755), whereas the AUC of the CF-corrected model was 0.692 (95% CI, 0.616-0.768), suggesting a modest improvement in discrimination after correction. DCA showed that the CF-corrected model provided slightly greater net benefit across part of the clinically relevant threshold probability range. However, the magnitude of this advantage was small and was not consistent across all thresholds. Continuous NRI analysis showed an event NRI of -0.250 (95% CI, -0.600 to 0.152), a non-event NRI of 0.530 (95% CI, -0.196 to 0.634), and a total NRI of 0.280 (95% CI, -0.368 to 0.673). Because the confidence intervals for all NRI components crossed zero, and the total NRI did not show a stable overall improvement, the overall reclassification benefit of correction was limited. The added value of this approach for clinical decision-making therefore requires validation in larger samples and external cohorts (**Figure 5**).

### 3.6 Prespecified clinical subgroup analyses

Because dd-cfDNA reflects graft injury rather than a single pathological process, we performed eight prespecified clinical subgroup analyses. These analyses had three aims: first, to assess whether the primary findings were consistent across different clinical contexts; second, to explore whether the dd-cfDNA signal was stronger or weaker in specific patient groups; and third, to identify potential effect modifiers for future multivariable models or more refined stratified analyses. It should be emphasized that subgroup analysis is not a method for controlling confounding and cannot replace multivariable regression. Its value lies in clarifying the clinical settings in which the observed association is more apparent, rather than proving that a given factor has been adjusted for.

**Figure 6:**
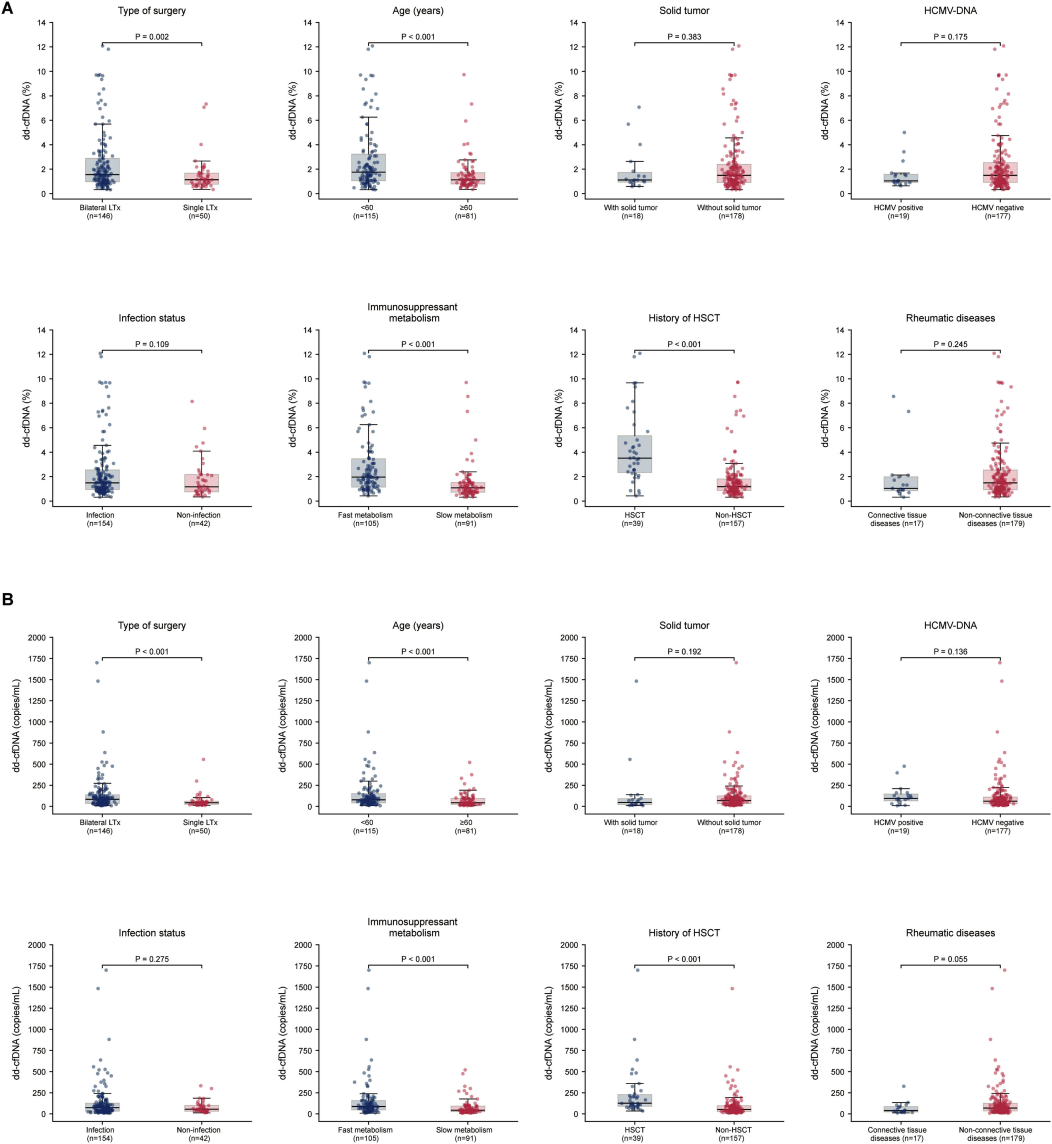
Subgroup analysis of **(A)** dd-cfDNA (%) and **(B)** dd-cfDNA (copies/mL) according to clinical characteristics.

**Figure 7:**
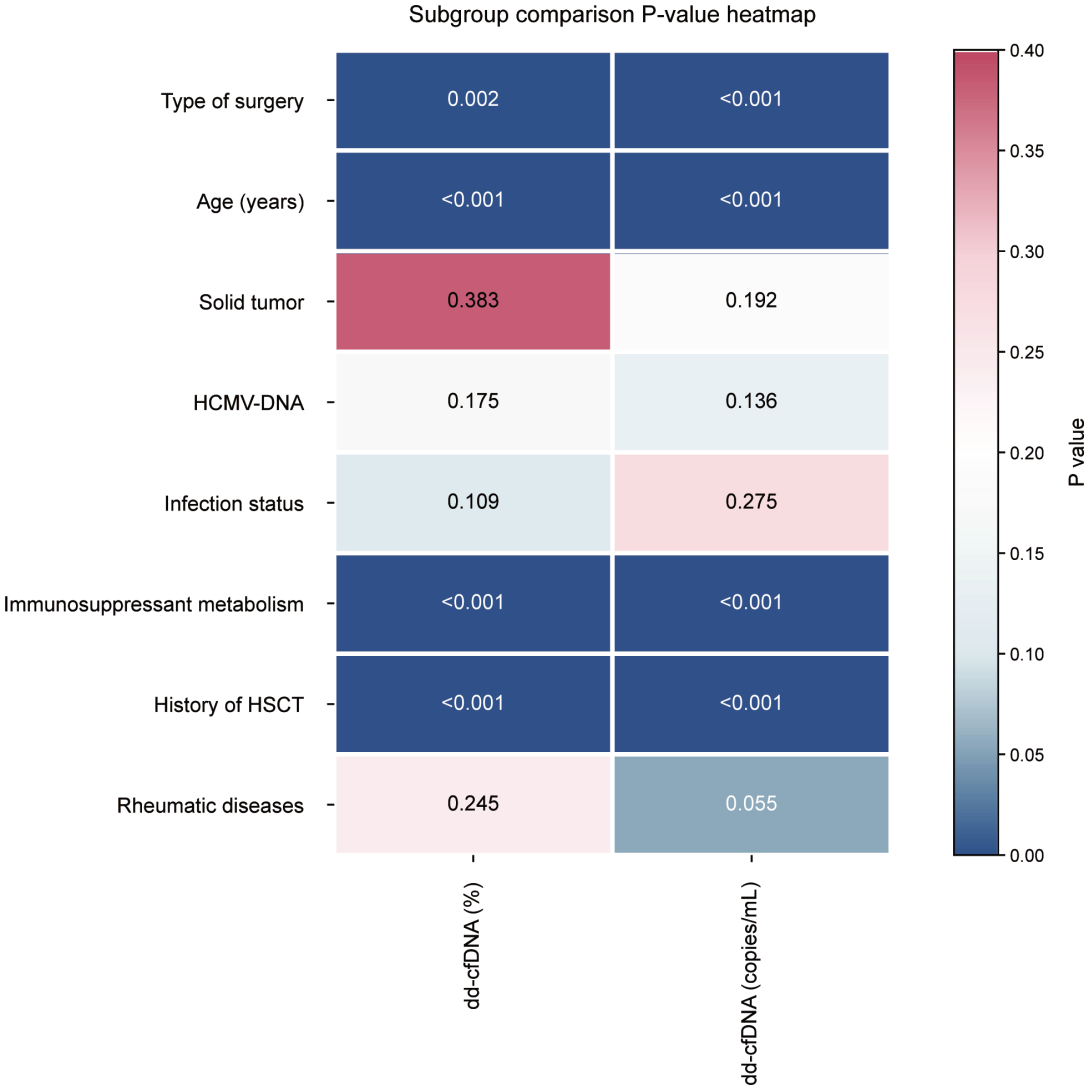
Heatmap of subgroup comparison P values for dd-cfDNA (%) and dd-cfDNA (copies/mL) according to clinical characteristics. The heatmap summarizes the P values for between-group comparisons across clinical subgroups, including type of surgery, age, solid tumor status, HCMV-DNA status, infection status, immunosuppressant metabolism, history of hematopoietic stem cell transplantation (HSCT), and rheumatic diseases. The color intensity reflects the magnitude of the P value, and lower P values indicate stronger evidence of subgroup differences in dd-cfDNA levels. This heatmap was used as a descriptive visualization of subgroup-specific statistical patterns rather than as a measure of feature importance or effect size.

The subgroup analyses showed that both dd-cfDNA (%) and dd-cfDNA (copies/mL) demonstrated more pronounced differences across strata defined by surgical procedure, age, immunosuppressant metabolism profile, and history of HSCT. Higher dd-cfDNA levels, together with lower corresponding P values, were observed in double-LTx recipients, patients aged <60 years, rapid immunosuppressant metabolizers, and patients with a history of HSCT. In contrast, infection status, HCMV-DNA positive, solid tumor, and rheumatic disease did not show consistent or statistically significant differences. The difference in dd-cfDNA (copies/mL) among patients with rheumatic disease was only borderline significant (P = 0.055) and did not reach statistical significance (**Table 5**; **Figures 6**, **7**).

**Table 5:** Subgroup comparisons of dd-cfDNA (%) and dd-cfDNA (copies/mL) based on clinical characteristics.

Characteristics	dd-cfDNA (%)	P value	dd-cfDNA (copies/mL)	P value
Type of surgery				
Bilateral LTx (n = 146)	1.56 (0.97-2.89)	0.002	84.01 (36.80-136.84)	<0.001
Single LTx (n = 50)	1.14 (0.77-1.67)		43.34 (26.21-61.05)	
Age (years)				
<60 (n = 115)	1.76 (1.06-3.24)	<0.001	78.85 (41.73-150.40)	<0.001
≥60 (n = 81)	1.13 (0.79-1.71)		43.56 (26.05-95.81)	
Solid tumor				
With solid tumor (n = 18)	1.12 (0.94-1.74)	0.383	47.33 (23.15-92.63)	0.192
Without solid tumor (n = 178)	1.50 (0.91-2.39)		68.79 (34.97-123.20)	
HCMV-DNA				
HCMV positive (n = 19)	1.05 (0.92-1.58)	0.175	95.81 (66.79-146.54)	0.136
HCMV negative (n = 177)	1.49 (0.92-2.53)		62.24 (31.31-109.68)	
Infection status*				
Infection (n = 154)	1.49 (0.94-2.55)	0.109	74.00 (32.61-126.00)	0.275
Non-Infection (n = 42)	1.17 (0.76-2.17)		55.06 (31.53-99.34)	
Immunosuppressant metabolism#				
Fast metabolism (n = 105)	1.96 (1.15-3.46)	<0.001	86.55 (46.97-157.30)	<0.001
Slow metabolism (n = 91)	1.08 (0.73-1.52)		43.56 (26.81-91.22)	
History of HSCT				
HSCT (n = 39)	3.51 (2.33-5.35)	<0.001	125.92 (89.85-227.27)	<0.001
Non-HSCT (n = 157)	1.18 (0.87-1.81)		52.30 (27.88-95.81)	
Rheumatic diseases				
Connective tissue diseases (n = 17)	1.04 (0.85-1.98)	0.245	38.90 (23.33-85.34)	0.055
Non-Connective tissue diseases (n = 179)	1.49 (0.93-2.54)		69.23 (35.06-125.47)	

Data are expressed as mean ± standard deviation, median (interquartile range), or *n* (%).

HSCT, hematopoietic stem cell transplantation.

*Infection status was defined as follows: If confirmed by metagenomic next-generation sequencing (NGS) or repeated body fluid culture, any one of bacteria, fungi or viruses (such as HCMV, EBV, etc.) is positive, then it is determined as “positive infection”.

^#^ Immunosuppressant metabolism type was mainly defined according to CYP3A5 rs776746 genotype test. CYP3A5*1/*1 and CYP3A5*1/*3, corresponding to rs776746 AA and AG genotypes, were classified as fast-metabolizer genotypes, whereas CYP3A5*3/*3, corresponding to the rs776746 GG genotype, was classified as a slow-metabolizer genotype.

## 4 Discussion

This single-center real-world cohort study evaluated dd-cfDNA as a noninvasive adjunct for identifying clinically diagnosed rejection-associated allograft injury after LTx. The main findings were as follows. First, both dd-cfDNA (%) and dd-cfDNA (copies/mL) were significantly higher in clinically diagnosed rejection than in non-rejection observations, but their diagnostic discrimination was moderate. Second, dd-cfDNA (%) remained associated with clinically diagnosed rejection after adjustment for key clinical variables and in sensitivity analyses accounting for repeated measurements. Third, dd-cfDNA levels differed systematically between single- and double-LTx recipients, and a stable-state correction factor improved numerical comparability, although its clinical utility remained limited and exploratory. Fourth, infection and other clinical factors should be considered when interpreting dd-cfDNA because dd-cfDNA reflects allograft injury rather than rejection specifically.

The moderate AUC values observed in this cohort should be interpreted in the context of real-world clinical complexity. Compared with studies using protocolized histopathological endpoints, our study used clinically adjudicated rejection as the primary reference standard, which may introduce misclassification because infection, CLAD, airway complications, and other non-alloimmune injuries can mimic rejection and may also elevate dd-cfDNA. In addition, the high infection burden, heterogeneous testing time points, inclusion of both single- and double-LTx recipients, and differences in assay methodology and endpoint ascertainment may have contributed to lower discrimination than that reported in some previous studies (17–19, 25–29). Therefore, our data support dd-cfDNA as an adjunctive marker for risk assessment rather than a stand-alone diagnostic test.

In this study, we focused specifically on infection instead of conducting separate exploratory analyses for all covariates, because infection is one of the most common complications after LTx and is often clinically difficult to distinguish from rejection (30). The present data suggest that infection may increase the background level of dd-cfDNA and may reduce the specificity of dd-cfDNA for rejection-associated injury. Although dd-cfDNA (%) remained associated with clinically diagnosed rejection after adjustment for infection status, our ability to separate the independent effects of infection and rejection was limited by the small rejection-only subgroup, the frequent coexistence of infection and rejection, and the pooling of different infection etiologies. Future studies should incorporate pathogen type, pathogen burden, infection severity, and time-aligned sampling relative to dd-cfDNA testing.

Another important source of heterogeneity is the composite nature of the rejection endpoint. Acute cellular rejection (ACR), antibody-mediated rejection (AMR), CLAD-related dysfunction, and mixed rejection phenotypes may differ in their underlying mechanisms, temporal evolution, histopathological features, and extent of allograft injury, and therefore may be associated with different dd-cfDNA release patterns. In the present retrospective cohort, however, phenotype-specific classification could not be reliably reconstructed because the required diagnostic elements were not uniformly available. This limitation may have contributed to the moderate discriminative performance observed in our study. Future prospective studies should incorporate standardized biopsy protocols, donor-specific antibody assessment, CLAD phenotyping, and time-aligned dd-cfDNA testing to clarify phenotype-specific dd-cfDNA dynamics.

Differences between single- and double-LTx represent a unique and important issue in dd-cfDNA research after LTx. In this study, we quantified the systematic effect of surgical procedure on dd-cfDNA levels and demonstrated both the need for and feasibility of a simple correction approach. This may help address a key barrier to harmonizing dd-cfDNA thresholds and standardizing clinical interpretation. Consistent with previous observations in single-LTx studies, we found that both dd-cfDNA (%) and dd-cfDNA (copies/mL) were lower in single-LTx recipients than in double-LTx recipients under both stable and clinical rejection conditions (21). This finding is biologically plausible because graft mass differs between single- and double-LTx recipients, which may affect both the total amount and relative proportion of donor-derived DNA released into the circulation (21, 25). Furthermore, the CF of 1.54 derived from stable-state values effectively reduced the numerical difference in dd-cfDNA (%) between single- and double-LTx recipients in the present cohort. However, it did not change the AUC within the single-LTx subgroup, indicating that the main value of correction is to improve cross-procedure comparability rather than to enhance ranking performance within single-LTx recipients. DCA and continuous NRI analyses showed a mild improvement in net benefit across part of the threshold probability range after CF correction, but this improvement was not consistent. The confidence intervals for each NRI component and for the total NRI crossed zero, suggesting that the correction strategy does not yet provide definitive evidence of improved clinical decision-making performance. Thus, this approach should be regarded as an initial standardization attempt to improve the numerical comparability of dd-cfDNA (%) between single- and double-LTx recipients, rather than as a fully validated clinical prediction model. Future studies may further refine individualized correction by incorporating imaging-based measurements of actual transplanted lung volume.

This study also used eight prespecified subgroup analyses to explore the clinical contexts in which the dd-cfDNA signal may be stronger. These results should be interpreted as supportive evidence for assessing the robustness and heterogeneity of the primary findings, rather than as new causal findings. Because multiple subgroup comparisons were performed without formal multiplicity adjustment, isolated statistically significant findings may represent false-positive results. Stronger dd-cfDNA differences were observed across strata defined by surgical procedure, age, immunosuppressant metabolism profile, and history of HSCT, suggesting that these variables may influence the background distribution or response magnitude of dd-cfDNA. In contrast, infection status, HCMV-DNA status, solid tumor, and rheumatic disease did not show consistent or statistically significant differences, indicating that, in the present cohort, they were more likely to contribute to background variability than to act as dominant effect modifiers. We further summarized this significance pattern using a heatmap, which visually demonstrated that the two dd-cfDNA measures did not show fully overlapping patterns of statistical significance across subgroups.

The subgroup difference according to immunosuppressant metabolizer phenotype deserves cautious interpretation. In this study, metabolizer status was pharmacogenetically defined, mainly according to CYP3A5 rs776746. CYP3A5*1 carriers are generally CYP3A5 expressers and may have lower dose-adjusted immunosuppressant trough concentrations than CYP3A5*3/*3 non-expressers. Therefore, if dosing is not adequately individualized, patients classified as fast metabolizers may have relatively lower effective calcineurin inhibitor exposure, which could theoretically contribute to a higher risk of rejection-associated allograft injury and increased dd-cfDNA release. However, our dataset did not include complete longitudinal immunosuppressant trough concentrations, concentration/dose ratios, adherence information, or time-varying dose adjustments for all observations. Therefore, the observed association between fast-metabolizer genotype and higher dd-cfDNA levels should be regarded as exploratory and hypothesis-generating rather than causal.

Finally, this study focused on quantitative dd-cfDNA measures only. Emerging cfDNA fragmentomic features, including fragment size profiles, fragmentation patterns, tissue-of-origin signatures, and end-motif characteristics, may provide additional biological information beyond donor fraction or absolute concentration. Integrating quantitative dd-cfDNA with qualitative cfDNA features may help improve specificity and distinguish rejection-associated injury from infection or other non-alloimmune causes of graft injury in future lung transplantation studies.

## 5 Limitations

The main limitations of this study are as follows. First, this was a single-center retrospective study with a limited sample size, and the timing of dd-cfDNA testing was not protocolized. Therefore, selection bias and residual confounding cannot be excluded. Second, systematic histopathologic confirmation was not available for all cases. Accordingly, this study used integrated clinical judgment as the primary reference standard. Although this approach reflects real-world clinical practice, some degree of misclassification remains possible. Third, infection was common in this cohort and frequently overlapped with clinically diagnosed rejection. Although infection status was included in regression and exploratory subgroup analyses, infection severity, pathogen burden, and the exact temporal relationship between infection and dd-cfDNA testing were not consistently available. Therefore, the independent effects of infection and rejection could not be fully separated. Fourth, the single-/double-LTx correction factor was derived and evaluated in the same cohort and should therefore be considered exploratory. External validation in independent cohorts is required before clinical implementation.

## 6 Conclusions

In conclusion, dd-cfDNA is a promising noninvasive adjunct for identifying rejection-associated allograft injury after LTx. Among the two measures, dd-cfDNA (%) appears better suited as a sensitive screening marker, whereas dd-cfDNA (copies/mL) may provide complementary specificity. Because dd-cfDNA reflects allograft injury rather than rejection specifically, infection status, transplant procedure, and other clinical causes of graft injury should be considered during interpretation. The stable-state correction factor may improve numerical comparability between single- and double-LTx recipients, but this exploratory approach requires external validation. Prospective multicenter studies with time-aligned histopathology, infection assessment, lung function trajectories, and clinical outcomes are needed to establish individualized dd-cfDNA-guided monitoring strategies.

## Data Availability

The datasets generated and/or analyzed during the current study are not publicly available because they contain potentially sensitive clinical information. De-identified data may be made available by the corresponding author on reasonable request, subject to institutional ethics approval and applicable data-protection requirements.

## Supplementary material

Supplementary Figure 1 Study design flowchart.

Supplementary Figure 2 Comparison of dd-cfDNA levels among stable, infection, and clinical rejection groups.
