# Supplementary material for: Donor-derived cell-free DNA testing for noninvasive monitoring of allograft rejection after lung transplantation
Source: Front Immunol. 2026 Jul 29;17:1895278. doi: 10.3389/fimmu.2026.1895278 (PMC13461464; doi:10.3389/fimmu.2026.1895278)
Supplement: Supplementary file 1 [file DataSheet1.pdf]

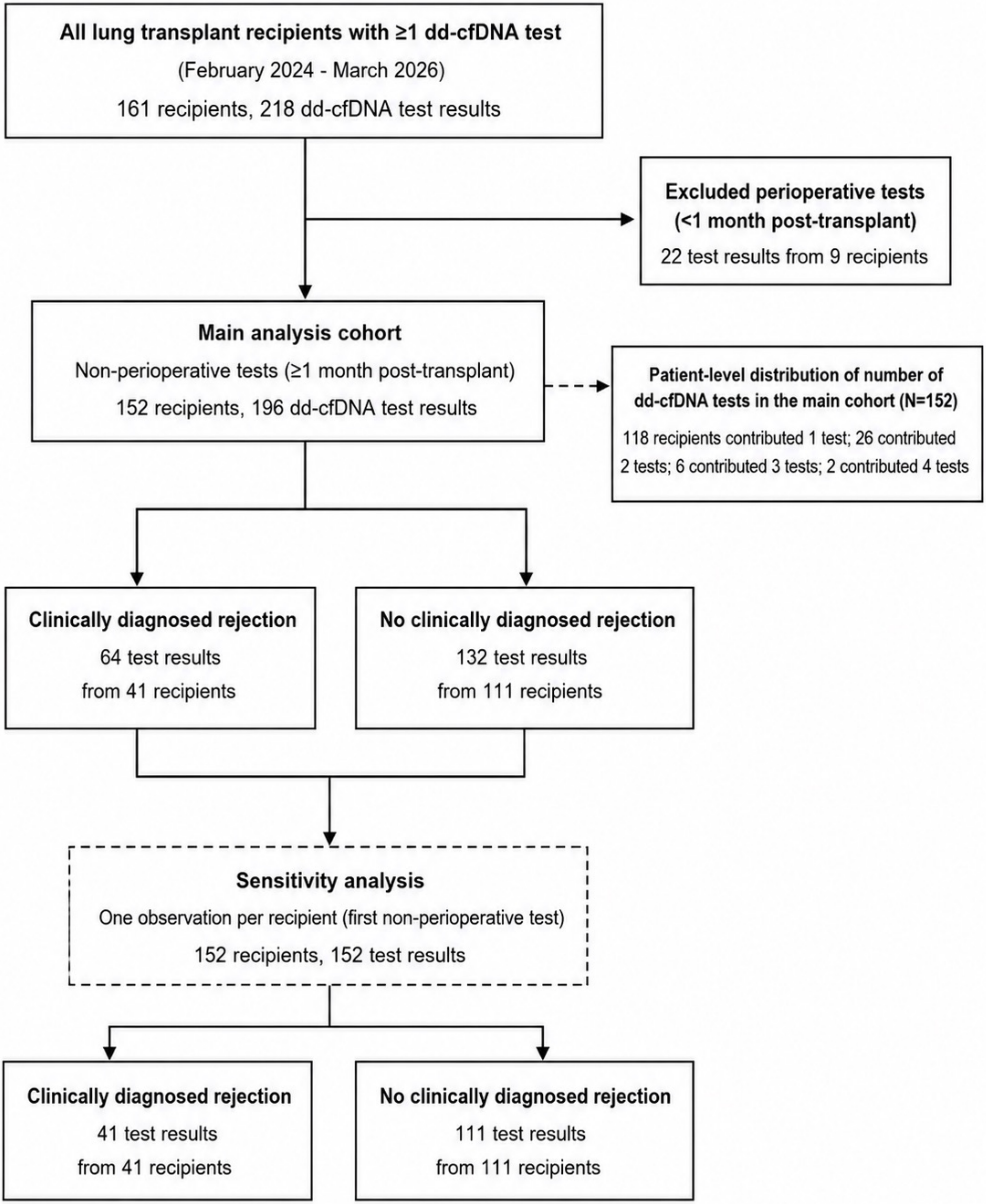
All lung transplant recipients with ≥1 dd-cfDNA test
(February 2024 - March 2026)
161 recipients, 218 dd-cfDNA test results
Excluded perioperative tests
(<1 month post-transplant)
22 test results from 9 recipients
Main analysis cohort
Non-perioperative tests (≥1 month post-transplant)
152 recipients, 196 dd-cfDNA test results
Patient-level distribution of number of dd-cfDNA tests in the main cohort (N=152)
118 recipients contributed 1 test; 26 contributed 2 tests; 6 contributed 3 tests; 2 contributed 4 tests
Clinically diagnosed rejection
64 test results
from 41 recipients
No clinically diagnosed rejection
132 test results
from 111 recipients
Sensitivity analysis
One observation per recipient (first non-perioperative test)
152 recipients, 152 test results
Clinically diagnosed rejection
41 test results
from 41 recipients
No clinically diagnosed rejection
111 test results
from 111 recipients
