# Supplementary material for: Donor-derived cell-free DNA testing for noninvasive monitoring of allograft rejection after lung transplantation
Source: Front Immunol. 2026 Jul 29;17:1895278. doi: 10.3389/fimmu.2026.1895278 (PMC13461464; doi:10.3389/fimmu.2026.1895278)
Supplement: Supplementary file 2 [file DataSheet2.pdf]

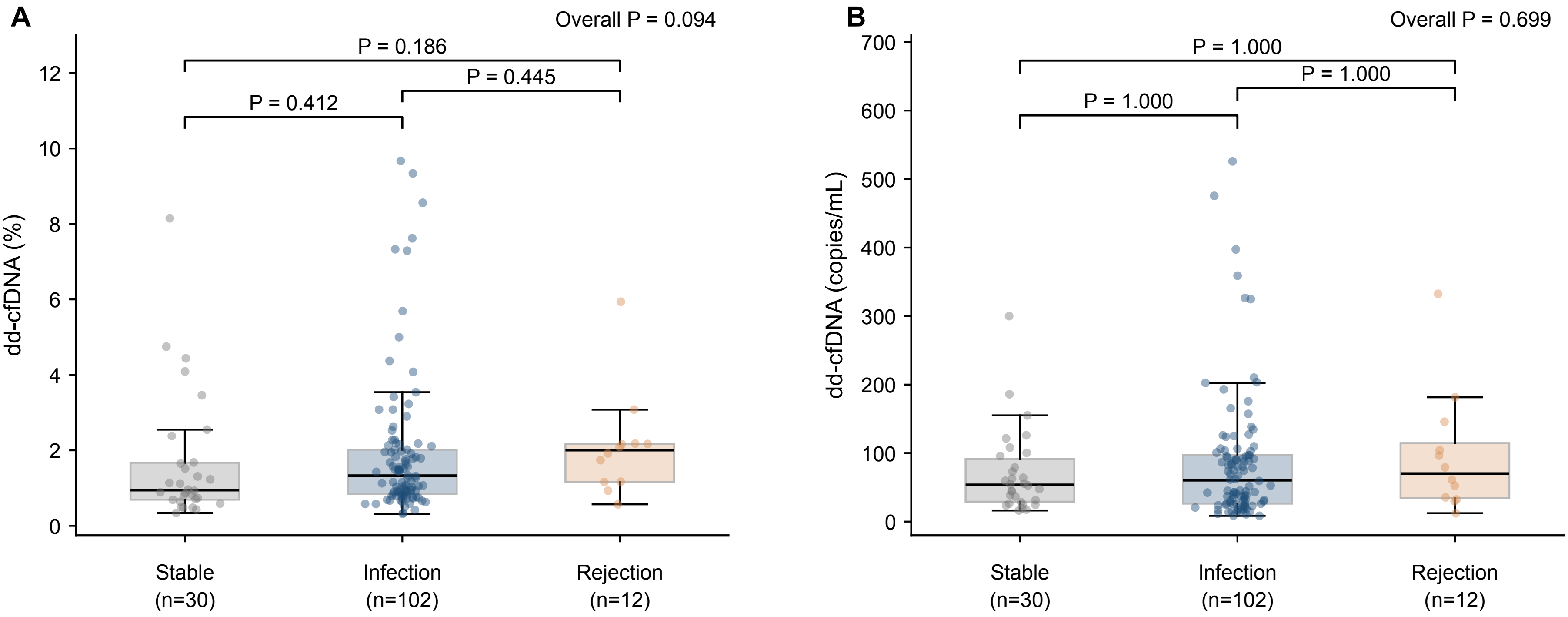
A
Overall P = 0.094
P = 0.186
P = 0.445
P = 0.412
dd-cfDNA (%)
0
2
4
6
8
10
12
Stable
(n=30)
Infection
(n=102)
Rejection
(n=12)
B
Overall P = 0.699
P = 1.000
P = 1.000
P = 1.000
dd-cfDNA (copies/mL)
0
100
200
300
400
500
600
700
Stable
(n=30)
Infection
(n=102)
Rejection
(n=12)
